# Genomic epidemiology of meticillin-resistant *Staphylococcus aureus* ST22 widespread in communities of the Gaza Strip, 2009

**DOI:** 10.2807/1560-7917.ES.2018.23.34.1700592

**Published:** 2018-08-23

**Authors:** Qiuzhi Chang, Izzeldin Abuelaish, Asaf Biber, Hanaa Jaber, Alanna Callendrello, Cheryl P Andam, Gili Regev-Yochay, William P Hanage, on behalf of the PICR Study Group

**Affiliations:** 1 Department of Epidemiology, Harvard T.H. Chan School of Public Health, Boston, United States; 2 Global Health Division, Dalla Lana School of Public Health, University of Toronto, Toronto, Canada; 3 Infectious Disease Unit, Sheba Medical Center, affiliated to the Sackler School of Medicine, Tel Aviv University, Ramat Gan, Israel; 4 Infectious Disease Epidemiology Section, the Gertner Institute for Epidemiology and Health Policy Research, Ramat Gan, Israel; 5 These authors contributed equally

**Keywords:** genomics, ST22, Staphylococcus aureus, MRSA, Gaza strip, phylogeny

## Abstract

Remarkably high carriage prevalence of a community-associated meticillin-resistant *Staphylococcus aureus* (MRSA) strain of sequence type (ST) 22 in the Gaza strip was reported in 2012. This strain is linked to the pandemic hospital-associated EMRSA-15. The origin and evolutionary history of ST22 in Gaza communities and the genomic elements contributing to its widespread predominance are unknown. **Methods:** We generated high-quality draft genomes of 61 ST22 isolates from Gaza communities and, along with 175 ST22 genomes from global sources, reconstructed the ST22 phylogeny and examined genotypes unique to the Gaza isolates. **Results:** The Gaza isolates do not exhibit a close relationship with hospital-associated ST22 isolates, but rather with a basal population from which EMRSA-15 emerged. There were two separate resistance acquisitions by the same MSSA lineage, followed by diversification of other genetic determinants. Nearly all isolates in the two distinct clades, one characterised by staphylococcal cassette chromosome *mec* (SCC*mec)* IVa and the other by SCC*mec* V and MSSA isolates, contain the toxic shock syndrome toxin-1 gene. **Discussion:** The genomic diversity of Gaza ST22 isolates is not consistent with recent emergence in the region. The results indicate that two divergent Gaza clones evolved separately from susceptible isolates. Researchers should not assume that isolates identified as ST22 in the community are examples of EMRSA-15 that have escaped their healthcare roots. Future surveillance of MRSA is essential to the understanding of ST22 evolutionary dynamics and to aid efforts to slow the further spread of this lineage.

## Introduction

Meticillin-resistant *Staphylococcus aureus* (MRSA) was first recognised as a threat in the healthcare setting, but since the early 2000s, it has been an increasing source of concern as a cause of community-acquired MRSA (CA-MRSA) infections [1]. These have been associated with outbreaks and attracted much attention as a cause of skin and soft tissue infections and severe invasive disease among young, otherwise healthy individuals [2]. However, the great majority of CA-MRSA transmission occur within the context of asymptomatic colonisation. Colonisation is important not only because it provides the opportunity for onward transmission but also because it is normally a prerequisite for infection and disease. Surveillance of healthy carriers is therefore a crucial part of understanding CA-MRSA epidemiology.

While the epidemiology of CA-MRSA has been well studied in the United States (US) and Europe, studies on CA-MRSA carriage and infection have been scarce in regions such as the Middle East. One of the highest recorded rates of CA-MRSA colonisation was previously reported in the Gaza strip, a 360-km^2^ narrow region located along the eastern Mediterranean coast populated by 1.7 million people. A single cross-sectional study conducted in 2009 by the Palestinian-Israeli Collaborative Research (PICR) group reported that nearly 15% of healthy individuals in Gaza neighbourhoods and villages carried MRSA [3], in stark contrast with the MRSA carriage prevalence in other geographical regions. Specifically, 10 studies reporting the prevalence of MRSA in the community in countries including Korea, Portugal, the United Kingdom (UK), and the US were analysed in a meta-analysis and the pooled data showed a prevalence of 1.3% for CA-MRSA carriage [4]. Another study in regions near Israel found that 0.15% of the study population carried CA-MRSA [5]. Molecular typing revealed that more than 70% of the isolates from Gaza were a single clone closely related to the epidemic MRSA-15 clone (EMRSA-15).

EMRSA-15 has been one of the most important clones causing disease in the healthcare setting over the last two decades. It typically owes its resistance phenotype to a type IVh staphylococcal cassette chromosome *mec* (SCC*mec*) element and is commonly referred to as sequence type (ST) 22 on the basis of multilocus sequence typing (MLST). However, MLST is based on the sequence of only seven housekeeping genes, a small fraction of the whole genome. This limitation can now be addressed with rapid and cost-effective approaches in next generation sequencing which provide high-resolution views of genomic variation in bacterial populations. A study of the genomic epidemiology of ST22 determined that EMRSA-15 belonged to a distinct healthcare-associated MRSA clade estimated to have originated in the early 1980s from a diverse founder population of community-associated isolates with the same ST that were mostly meticillin-susceptible *S. aureus* (MSSA) [6]. The EMRSA-15 clone has been termed ST22-A to distinguish it from the community-acquired ancestral non-ST22-A populations that are indistinguishable by MLST and that are very rarely meticillin-resistant [6]. Whether the ‘Gaza clone’ is descended from ST22-A, having readapted to the community from the hospital niche, or is an independently emerging successful clone from the same ancestral population that gave rise to ST22-A, is unknown. Using high quality draft genomes, we have examined the evolution of this particular strain and its phylogenetic relationship to the EMRSA-15 clone, as well as identified genes that may drive its success in the community setting.

## Methods

### Bacterial isolates

The study population from which the *S. aureus* strains were isolated has been previously described [3]. Briefly, healthy children younger than 5.5 years were randomly selected from 12 Gaza neighbourhoods and villages in the northern and central Gaza strip between March and July 2009. Following parental signed informed consent, children and one of their parents were sampled with nasal swabs. Institutional review board approval was given by the Sheba Medical Center as well as by a local ethics committee of the Health Ministry in the Gaza strip. In total, 61 ST22 isolates were included in this study, namely 53 MRSA and six MSSA isolates collected in Gaza. As comparison, we also included one MRSA isolate collected from the neighbouring city of Bat Yam, Israel and an EMRSA-15 strain from the UK for sequencing. *Spa*-typing of the isolates was performed as previously described [3]. Details of the collection are provided in Supplement 1. Sequence data have been deposited in the Sequence Read Archive under study number SRP156559 (https://www.ncbi.nlm.nih.gov/sra).

### DNA extraction and sequencing

DNA was extracted and purified from cultures using DNeasy Blood and Tissue kit (Qiagen, Valencia, US). DNA libraries were prepared using the NexteraXT protocol (Illumina, San Diego, US) with 1 ng of genomic DNA per isolate. Samples were sequenced as multiplexed libraries on the MiSeq platform (Illumina, San Diego, US) to produce paired-end reads of 150 nt in length. Summary assembly statistics including quality scores and coverage are presented in Supplement 1. The ST of each isolate was confirmed using the programme Short Read Sequence Typing (SRST2) [7].

### Phylogenetic analysis

Both de novo and reference-based genome assemblies were constructed. The reads for each isolate were assembled de novo using the short-read assembler Velvet v1.2.10, with summary statistics shown in Supplement 2, and the resulting contigs were annotated using Prokka [8]. Pan-genome analysis was performed using Roary v3.6 [9]. For reference-based assemblies, paired-end Illumina reads for each isolate were mapped onto the *S. aureus* ST22 reference genome HO 5096 0412 (GenBank accession number: HE681097) using SMALT version 0.7.6 (http://www.sanger.ac.uk/science/tools/smalt-0), giving on average 30× depth of coverage for more than 93% of the reference genome. The resulting read alignment was processed using SAMtools [10] and VCFtools [11]. Sites with an abnormally high single nucleotide polymorphism (SNP) density were detected and removed using Gubbins [12].

We screened all genomes for known resistance and virulence genes using a direct read mapping approach implemented in SRST2 [7]. BLASTN searches were also performed to compare de novo assemblies against databases of *S. aureus* genes associated with antibiotic resistance [13] and virulence [14], which were compiled from literature and public sequence databases. An e-value of 10^−10^ was used for identifying top BLAST hits.

A maximum likelihood phylogenetic tree was constructed using RAxML v.8.1.15 [15] using a generalised time reversible (GTR) model with a gamma correction for among-site rate variation. To determine how the Gaza isolates are related to the worldwide population of ST22, and in particular the pandemic EMRSA-15/ST22-A lineage, the Gaza genomes were compared with a previously published collection of 175 genomes from a global collection of ST22 *S. aureus* [6]. This collection consists of 147 ST22-A isolates mostly of hospital origin, and 28 non-ST22-A isolates which were more diverse, usually MSSA and of community origin.

To date the origin of the Gaza clade, we first investigated the temporal signal in the recombination-free phylogeny generated by Gubbins using TempEst v1.5.4 [16]. When no signal was evident, which prevented an estimation of a clock rate from these data, we applied a strict molecular clock to estimate the time to the most recent common ancestor (tMRCA) using BEAST v1.8.2 [17]. We used the evolutionary rate previously determined for the non-ST22-A clade of 1.48 x 10^−6^ substitutions per nucleotide site per year, adjusted for the number of SNPs observed. We ran the analyses under two parametric (constant population size and exponential growth) and one non-parametric (Bayesian skyline plot) coalescent tree priors. Both Hasegawa-Kishino-Yano (HKY) and GTR nucleotide substitution models were used, and non-uniformity of evolutionary rates among sites was modelled using a discrete gamma distribution with four rate categories. All other default parameters in the programme BEAUti v1.8.2 were used [18]. For each analysis, we ran the Markov chain Monte Carlo (MCMC) chain for 100 million generations, with sampling every 10,000 generations. The initial 10% of the samples from the beginning of each run were treated as burn-in and removed from the analysis. Output for each chain was checked using Tracer (http://tree.bio.ed.ac.uk/software/tracer/) to ensure that effective sample size values for all parameters were greater than 200. The maximum clade credibility tree was generated using TreeAnnotator v1.8.2 as implemented in BEAST (http://beast.bio.ed.ac.uk/) and visualised using FigTree (http://tree.bio.ed.ac.uk/software/figtree/).

## Results

We sequenced 53 MRSA and six MSSA isolates from Gaza as well as one isolate from Bat Yam, Israel and one EMRSA-15 strain from the UK. Among the 59 isolates from Gaza, a total of 2,232 SNPs in the core genome were found. The *spa* type of all 59 isolates from Gaza was t223, which has been found in numerous European and Middle East countries with a global frequency of 0.49% (http://spa.ridom.de/frequencies.shtml). The characteristics of the Gaza collection are summarised in Supplement 1.

**Figure 1:**
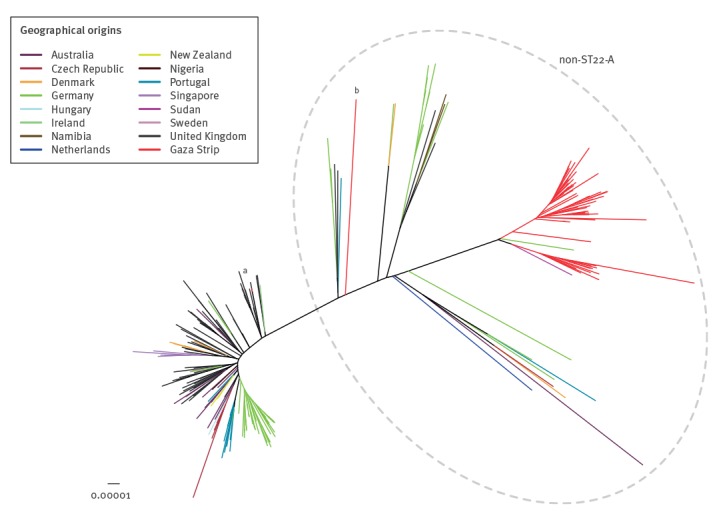
Unrooted maximum likelihood core genome tree of meticillin-resistant *Staphylococcus aureus* ST22, Gaza, 2009 (n = 59)

Overall, 6,944 SNPs found in the core genome of 236 isolates, including the 61 isolates included in the study and 175 ST22 genomes from global sources, were used to construct the maximum likelihood tree shown in Figure 1. All ST22 isolates from Gaza grouped with the diverse non-ST22-A population, the majority of which were community-acquired. The EMRSA-15 strain from the UK grouped accordingly with healthcare-associated ST22-A isolates as expected. In addition, the only isolate in our sample collected in Israel grouped elsewhere in the non-ST22-A population.

The ST22 Gaza population was compared with 175 isolates representative of the global ST22 population [6], based on 6,944 SNPs. The ST22 population was further divided into the ST22-A population, the majority of which were of healthcare origin, and the non-ST22-A population, the majority of which were of community origin. Branches are coloured according to their geographical origin.

^a^EMRSA-15 strain from UK sequenced in our laboratory.

^b^ST22 isolate from the Israeli city of Bat Yam.

**Figure 2:**
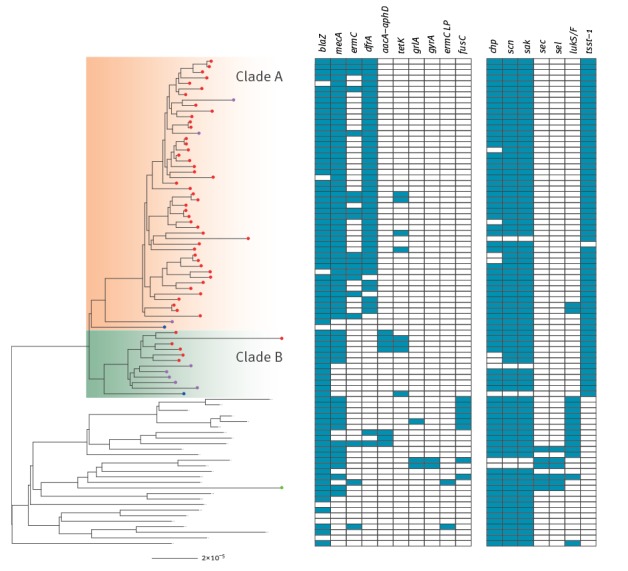
Presence and absence of genes involved in antibiotic resistance and virulence for meticillin-resistant *Staphylococcus aureus* ST22 non-ST22-A populations, Gaza, 2009 (n = 88)

MRSA: meticillin-resistant Staphylococcus aureus; MSSA: meticillin-sensitive Staphylococcus aureus.

The maximum likelihood tree in Figure 2 illustrates the phylogenetic relationship among the sampled isolates as well as with the other globally representative non-ST22-A isolates. The Gaza isolates formed two distinct clades, which we refer to as clade A and clade B. Clade A contained the majority of Gaza isolates (48/59), which all carried SCC*mec* IVa, and was closely related to an MSSA isolate from Gaza. Clade B consisted of six MRSA isolates with SCC*mec* V and was closely related to the majority of the MSSA isolates in our collection (5/6). Hence, these results suggested that the ‘Gaza clone’ is actually two distinct lineages resulting from two separate acquisitions of the SCC*mec* element.

Left panel: midpoint-rooted maximum likelihood phylogenetic tree of Gaza isolates along with non-ST22-A isolates. Red tips: MRSA isolates from the Gaza region; purple tips: MSSA isolates from the Gaza region; green tip: strain from Bat Yam, Israel; blue tips: the two closest non-ST22-A isolates from Germany and Sudan.

Right panel: presence (blue) or absence (white) of genes.

Resistance genes: *blaZ* (penicillin), *mecA* (oxacillin), *ermC* (erythromycin), *dfrA* (trimethoprim), *aacA-aphD* (gentamicin), *tetK* (tetracycline), *grlA* and *gyrA* (fluoroquinolone), *ermC* LP (clindamycin), *fusC* (fusidic acid).

Toxin genes: *chp* (CHIPS), *scn* (SCIN), *sak* (staphylokinase), *sec* (enterotoxin C), *sel* (enterotoxin L), *lukS/F-PV* (Panton–Valentine leukocidin), *tsst-1* (toxic shock syndrome toxin-1).

*S. aureus* has been found to contain multiple mobile genetic elements, some of which have been implicated in virulence or antibiotic resistance (such as SCC*mec*). The distribution of these genes between the two clades is summarised in Figure 2 and compared with the genes previously found in the non-ST22-A basal population. The *blaZ* gene encoding penicillin resistance was found on a Tn-554-like transposon which three isolates from Gaza appeared to have lost. Resistance to erythromycin encoded by *ermC* was found in 16 of 47 MRSA isolates restricted to clade A. In contrast, resistance to gentamicin encoded by the *aacA-aphD* gene was found only in clade B among four of five MRSA isolates. Tetracycline resistance encoded by the *tetK* gene was found in seven of 59 isolates in both clades. The distribution of these genes on the phylogeny indicates multiple acquisitions unique to each clade. In addition, almost all clade A isolates, and none of the clade B isolates, harboured the Tn4003 transposon carrying the *dfrA* gene, encoding resistance to trimethoprim.

We found resistance loci in other non-ST22-A strains that were not present in either clade of the Gaza isolates (Figure 2): a deletion in the *erm*C leader peptide region (clindamycin), *fusC* (fusidic acid) and mutations in *grlA* and *gyrA* genes predicted to give rise to fluoroquinolone resistance, which is characteristic of the EMRSA-15 expansion. All Gaza isolates had a small deletion of 372 bp in the *fnbA* gene and a large deletion of 2,464 bp in the *fnbB* gene, resulting in a gene fusion that retained most of the *fnbA* gene. This gene fusion was not found in the isolate from Bat Yam, Israel.

Similar to other non-ST22-A isolates, most of the Gaza isolates carried the prophage ΦSa3, which encodes staphylokinase [19], and the immune evasion proteins CHIPS and SCIN [20]. However, as shown in Figure 2, the prophage in some isolates was missing the *chp* gene which encodes the chemotaxis inhibitory protein and may play an important role in human immune invasion [20]. Other virulence markers of interest included Panton–Valentine leukocidin (PVL), which has been associated with severe skin infections and necrotising pneumonia, and TSST-1, the principal cause of staphylococcal toxic shock syndrome. The *lukS-PV*/*lukF-PV* genes encoding PVL were present in two clade A isolates but otherwise absent from our samples. However, all but one of the Gaza isolates harboured the *tsst-1* gene. This gene, found on a pathogenicity island, was ubiquitous in both Gaza clades and also the two most closely related non-ST22-A MSSA from the global collection but not in any others, indicating that it is characteristic of this Gaza lineage. As noted above, the isolate from Bat Yam, Israel was not found to be closely related to the Gaza isolates; it contained a pathogenicity island encoding enterotoxin C [21] and the *sel* gene encoding enterotoxin L that has been previously found in several members of the non-ST22-A population. However, these genes were absent from all of the Gaza isolates.

**Figure 3:**
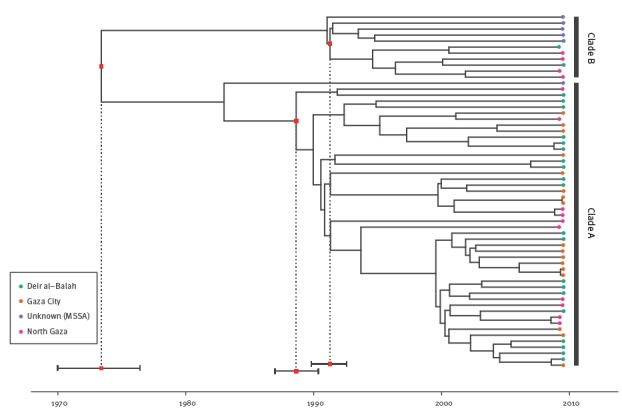
Maximum clade credibility tree of the meticillin-resistant *Staphylococcus aureus* ST22 population based on BEAST analysis using a strict molecular clock, Gaza, 2009 (n = 59)

To further investigate the evolutionary history of the ST22 Gaza lineage, Bayesian phylogenetic reconstruction was used to explore the temporal and geographical spread of the Gaza isolates (Figure 3). Isolates from different districts were intermingled. Bayesian analysis using a HKY substitution model and a Bayesian coalescent skyline as a tree prior showed that the time of the most recent common ancestor (MRCA) of the MRSA ST22 Gaza lineage was ca 1973 (95% highest posterior density (HPD) interval: 1970–76). However, the MRCA of clade A isolates probably emerged in 1989 (95% HPD: 1987–90), while the MRCA of clade B isolates originated in 1991 (95% HPD: 1990–93). Alternative substitution models and coalescent tree priors for the Bayesian analysis resulted in very similar tMRCA (Supplement 3).

MSSA: meticillin-sensitive Staphylococcus aureus.

Molecular clock: 1.48×10^−6^ substitutions per nucleotide site per year. Tips of the tree are constrained by isolation dates; the time scale is shown below the tree. The coloured tips of the tree indicate the geographical district in which the isolate originated. Dotted lines indicate time of the most recent common ancestor and the intervals indicate the 95% highest posterior density intervals.

## Discussion

The *S. aureus* lineage ST22 is known primarily as the genotype of the healthcare-associated EMRSA-15 clone; however, recent studies report of its increasing existence in carriage populations and its association with disease in community settings [22,23]. Previous work found CA-MRSA ST22 to be especially common in the Gaza strip, for reasons that are unknown [3]. In this study, we have sequenced the genomes of 59 isolates from Gaza and compared them to a previously described sample of ST22. While the Gaza strip is considered as one of the most densely populated regions in the world, there is little information on the epidemiology, diversity and evolution of *S. aureus* and other pathogens. To our knowledge, this is the first study to examine the population genomics of CA-MRSA in the Middle East, and in particular the Gaza strip.

Based on genomic data, we were able to resolve that the isolates previously described as the ‘Gaza clone’ [3] are actually two divergent clones, resulting from two independent acquisitions of SCC*mec* elements, rather than the gain of one element and then replacement by the other in descendants of that strain. One clone was produced by acquisition of SCC*mec* IVa and the other by SCC*mec* V, which provides a simple means of distinguishing them. Moreover, both Gaza clones emerged from the diverse founder ST22 population that is made up mostly of susceptible isolates of community origin and from which EMRSA-15 originated. EMRSA-15, by contrast, is a tight, closely related group of healthcare-associated isolates which has previously been termed ST22-A. Our phylogenetic analyses indicate that the Gaza isolates did not evolve from healthcare-associated MRSA that spread into the community, but rather from susceptible isolates. This was supported by the observation that the MSSA isolates included in this work, which were also collected in Gaza, branched off at the base of the two MRSA lineages. These results imply that researchers should not assume that isolates identified as ST22 in the community are examples of EMRSA-15 that have escaped their healthcare roots.

Clock rates have been independently reported for ST22 lineages and we used these to date the origins of the clones [6]. We estimated that the MRSA isolates in clade A had a common ancestor in the late 1980s and the MRSA isolates in clade B a few years later. EMRSA-15 isolates (termed ST22-A2) were previously estimated to have emerged from an earlier population (ST22-A1) around the same time period. With due caveats about the fact that our result depends on the clock rate, this suggests that this CA-MRSA lineage is older than might have been expected, given that CA-MRSA were initially reported as a major cause of concern in the early years of the 21st century [24].

The success of MRSA ST22 in the Gaza strip, in the community setting, cannot be attributed to a single cause and instead varies between the two major clades. The lineage we term clade A is notable for the almost universal presence of the *dfrA* gene encoding trimethoprim resistance and multiple acquisitions of the *ermC* gene encoding erythromycin resistance. The multiple gains and losses of the *ermC* gene are reminiscent of the history of EMRSA-15. However, the scattered distribution of the *ermC* gene within clade A suggests that resistance is easily acquired but not maintained and may indicate that this locus incurs a fitness cost. These two genotypes are completely absent from MRSA isolates in clade B which instead shows resistance to gentamicin. In addition, the presence of the *fnbA*-*fnbB* gene fusion and *tsst-1* gene in all Gaza isolates may have contributed to its spread in the region. FnBPA and FnBPB play a crucial role in binding to components of the host extracellular matrix proteins fibronectin, fibrinogen, and elastin, an important mechanism for colonisation of host tissues during infection [25]. Such gene fusions between fnbA and fnbB were not previously found in the non-ST22-A founder population, but have been previously reported in the EMRSA-15/ST22-A clade [6]. The consequences of these properties are not clear, but it is possible that they contribute to the increased transmissibility of ST22 isolates in Gaza communities.

The reasons for the extremely high prevalence of MRSA community carriage in Gaza remain unknown and may be a combination of risk factors in the community and properties of the strain. The current work cannot answer this question, but we suspect that the MSSA lineage that gave rise to these clones is successful and widespread beyond Gaza. This is because its closest relatives from the global collection were MSSA isolated in Germany and Sudan. This is insufficient evidence that the clones found in the Gaza strip originated in either of those countries. In fact, the long branch lengths between each clade and MSSA isolates from Germany and Sudan suggest a relatively distant common ancestor. Their widespread geographical distribution also suggests that this MSSA lineage, which is distinguished primarily by the presence of the *tsst-1* gene, has been highly successful outside healthcare settings.

MRSA in the community constitutes an important and still evolving public health challenge. While Europe has in general experienced a relatively low prevalence of CA-MRSA [26], the continent also exhibits considerable geographical variation in the prevalence of resistance across multiple bacterial pathogens. The Gaza clones described here may pose a threat to the status quo, as there is evidence these clones may be present, and increasing in prevalence, beyond the Gaza strip. Even though the *S. aureus* isolates from Gaza were sampled in 2009, recent epidemiological studies in countries such as Egypt, Italy, Jordan, Kuwait and Saudi Arabia have reported isolates that share many of the features of the lineages we have described in this work, such as the presence of SCC*mec* IVa and the *tsst-1* gene [27-31]. The presence of the *fnbA*-*fnbB* gene fusion we have identified in this work may be helpful in identifying other members of the lineages studied here, but without more data, ideally genomic data, it is not possible to determine the precise relationship between these isolates from other locations and the Gaza clones. However, the presence of shared features, such as the *tsst-1* gene, and a relatively widespread *spa* type (t223) suggests that they may be related and that these clones are widespread in the Mediterranean and Middle East regions and perhaps beyond. Future surveillance of MRSA in the community is essential to understand ST22 evolutionary dynamics and to aid efforts to slow the further spread of this lineage. This work shows that ST22 MRSA should not only be considered as a spillover from healthcare settings but potentially as a common component of the bacterial population in the community. Furthermore, it may be harbouring important virulence loci such as *tsst*-1.

Additional studies are needed to understand how these ST22 carriage isolates observed in the Gaza community are related to clinical strains in the region. A recent cross-sectional study to describe MRSA in the primary hospital in Gaza found that MRSA represented 56% of all clinical *S. aureus* isolates, the most prevalent of which was ST22 harbouring the *tsst-1* gene resembling the Gaza clones [32]. In our study, we lacked clinical isolates from Gaza to determine the direction of introduction of this particular strain. It is possible that the high levels of community carriage of the ST22 lineage serve as a reservoir driving its prevalence in the nosocomial setting, or vice versa. Future work should be directed at understanding where the selective pressures that drive the emergence and persistence of resistance are most important: Do we experience disease in the community arising from antibiotic selection in healthcare, or is antibiotic use in the community the key factor in this emerging problem? Genomic sequencing of additional MRSA ST22 isolates from other regions and of clinical isolates will improve our understanding of the evolution and prevalence of the Gaza lineage.
